# On the Theory of the Formation of Valvular Diseases of the Heart

**Published:** 1851-11

**Authors:** John Simon


*On the Theory of the Formation of Valvular Diseases of the Heart.* By John Simon, Esq., F.R.S.—Mr. Simon regards fibrin not as the nutritive material of the blood, but, with Zimmerman and other physiologists, as the result of the waste of the tissues, or the decay of the blood itself. We may ask, if this is so, why is the quantity of chyle greatest in the thoracic duct, having appeared gradually during the progress of the chyle from the lacteals? No doubt many evils result from excess of fibrin in the blood, as fibrinous concretions in the spleen, liver and kidneys. Mr. Simon even supposes the fibrinous vegetations, on the valves of the heart, so often found in acute rheumatism, and attributed to endocarditis, are owing to this cause; and he asks the important question—How do those valve diseases originate? He answers it as follows:

The general opinion, which till very lately has been unquestioned, is, that the lining membrane inflames, and pours out lymph, (just as the pleura or pericardium might do,) and that thus these vegetations are true *excrescences*, which have arisen in an inflammatory exudation.

Now here, on the threshold of the subject, a doubt occurs—Is the lining membrane of the arterial system susceptible of inflammation?

"Inflammation," in the language of the schools, "consists in an increased action of the bloodvessels of a part." Without pledging ourselves for the accuracy of this definition, we may use it in the present instance as a rough criterion of inflammation; at least negatively. No part can be said to inflame, in the ordinary sense of the word, which has not vessels of its own. You cannot talk of inflammation of the hair or nails. What, then, are the bloodvessels of the lining membrane of the arterial system, which this theory supposes to be the seat of an increased action? That portion of an artery which we term its inner coat, has no bloodvessels of its own, nor do those of the middle or contractile coat (which are derived from the so-called *vasa vasorum*) penetrate to a sufficient depth to influence materially (if at all) the nutrition of the lining membrane.

In the Museum of the College of Surgeons there is a preparation of Mr. Quekett's (an injection done, as all Mr. Quekett's are, with admirable success), which shows how very much the *vasa vasorum* fall short of reaching the lining membrane of the artery. And, if you will but think for a moment, what should *vasa vasorum* do there? what could they convey more fit for the nutrition and growth of the membrane than what is already there?—than that blood, namely, which is already washing over it incessantly and profusely.

And again, gentlemen, reflect on this: the lining membrane of the artery and of the valves has an epithelium; wherever epithelium grows, the old cells drop off in a direction opposite to that whence the new ones derive their materials of growth. Just as our scales of epidermis drop from the surface of our bodies, being nourished and renewed from within, just so, if the epithelium of an artery were renewed from without (*i. e.* by *vasa vasorum,*) the old scales, as fast as they became detached, would drop into the interior of the vessel; and though, no doubt, their decay is very slight, and the number that might thus pass into the circulation, and presently obstruct the capillaries, would be very small, still it would be essentially a clumsy arrangement.

From a variety of reasons, it seems almost certain that the ordinary nutrition of the lining membrane of the circulating system is derived directly from the blood with which it has contact; and that its morbid changes depend—not on any inflammatory condition due to the *vasa vasorum*, but on those humoral changes, those variations in the qualities of the blood, by which it is more immediately and more certainly affected than any tissue of the body.

On these grounds there would be great *primâ facie* difficulty in believing that the endocardial deposits could be of inflammatory origin. But this is not all; for if they were inflammatory exudations, why should they be so peculiarly limited to projecting or uneven surfaces of the lining membrane? And why should they evince so decided a preference for the *left* side of the heart? Both sides of the heart, and all points of each cavity, are, (one would think) equally exposed to the causes of inflammation; the coronary arteries supply both ventricles of the heart indifferently, and we well know that acute *peri*-carditis pays no respect to the grooves and septum of the heart; it traverses all such lines of demarcation, injects the blood-vessels of right and left side alike, and covers ventricles and auricles equally with its dense inflammatory exudation. On the supposition that these vegetations are inflammatory effusions from the membrane, I should be quite unable to explain why they should almost entirely confine themselves, as they do, to the valvular apparatus; and why their predilection for the left side should be so great, that the right is very rarely affected—perhaps never, except where the left has first suffered, and where the disease has been of such extreme intensity, that even its weaker affinity for the right side has been able to manifest itself.

The opposite doctrine is the more tenable one. I believe that the origin of these vegetations is directly humoral; that they arise as fibrinous precipitations from an overcharged solution; the valves encrusting themselves with fibrin, just as a stick in certain streams coats itself with a calcareous envelope; and that the preference shown for the left side of the heart admits of explanation by reference to the peculiarities of its contents—the new-made arterial blood.

You will observe that this theory involves the supposition, that arterial blood is more prone than venous blood to precipitate its fibrin, either as containing more of it, or as containing it in some more separable form.

Not wishing to leave this matter of uncertainty, I have experimented on the subject. I have carried a single thread, by means of a very fine needle, transversely through the artery and vein of a dog, leaving it there so that it might cut the stream; and I have done this repeatedly, sometimes in the femoral vessels, sometimes with the carotid and jugular, sometimes with the aorta and cava. I have suffered the thread to remain during a period of from twelve to twenty-four hours. My experiments have given me as a uniform result, that the arterial blood with the utmost readiness deposits its fibrin on the thread; the venous blood with the utmost reluctancy. And in most of my experiments, the thread, where it traversed the canal of the artery, presented a very considerable vegetation on its surface (exactly like those we are talking of on the valves of the heart;) a vegetation sometimes as large as a grain of wheat; always of a pyramidal shape, with its apex down-stream, and its base attached to the thread. In the artery, one might say that the thread whipped the blood, just as one whips blood in a basin to get the fibrin out of it; but with this trifling difference, that, instead of the rod beating the fluid, the fluid ran over the rod and precipitated its fibrin there. In the vein the thread seemed to operate no way but obstructively; never coating itself with fibrin, but sometimes delaying or stopping the circulation with a voluminous black clot, chiefly collected on that side of the thread remotest from the heart. Accordingly, the general statement and rationale of the matter appears to be as follows: The disease in which these deposits are so frequent is one of intense over-fibrination of the blood, and one in which almost certainly there are other conditions, besides *quantity*, making the fibrin easy of precipitation; the left side of the heart has preference, because it is the arterial side, and because arterial blood, as we have seen, readily parts with its fibrin; the valves, and particularly their streamward surfaces, are chosen for the deposit, because their position exposes them chiefly to the friction of the current; so that the whole curious selection of site for the deposit resolves itself into the concurrence of two conditions, which are fulfilled in that one spot of the vascular system—namely, the greatest chemical tendency to the deposition of fibrin, with the greatest mechanical facilities for its entanglement.

In introducing this subject, I mentioned that it is one of great practical importance. Many people bleed locally, or even generally, when they hear an endocardial murmur arising in the course of rheumatic fever. In their eyes, the new disease is endocarditis; and everything ending in *itis* is thought, in at least a majority of instances, to be benefitted by bleeding. Therefore do not be in a hurry to call it endocarditis; and as for bleeding, all that I would venture to say (for of course the treatment of this physician's disease does not fall within my province) is to assure you of the pathological fact, that you may bleed a patient to death without altering (except probably to increase) the proportion of fibrin in his blood.—*London Journal of Medicine.*

